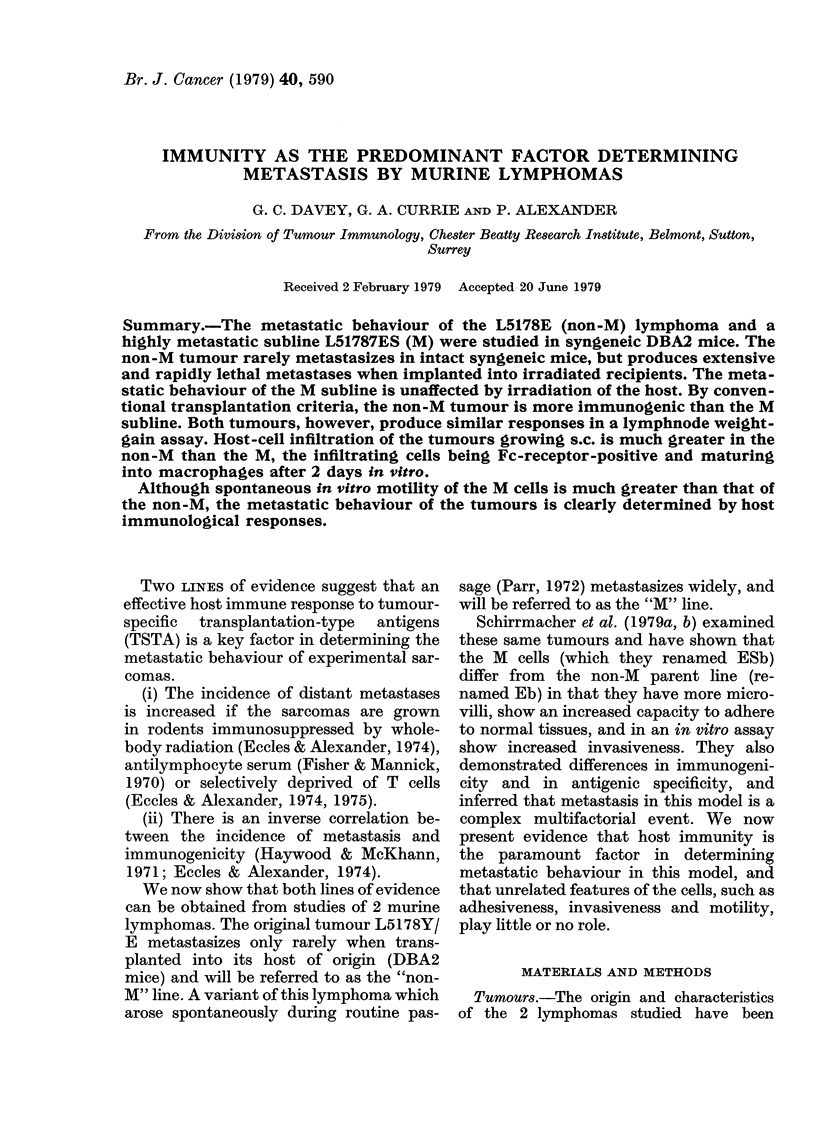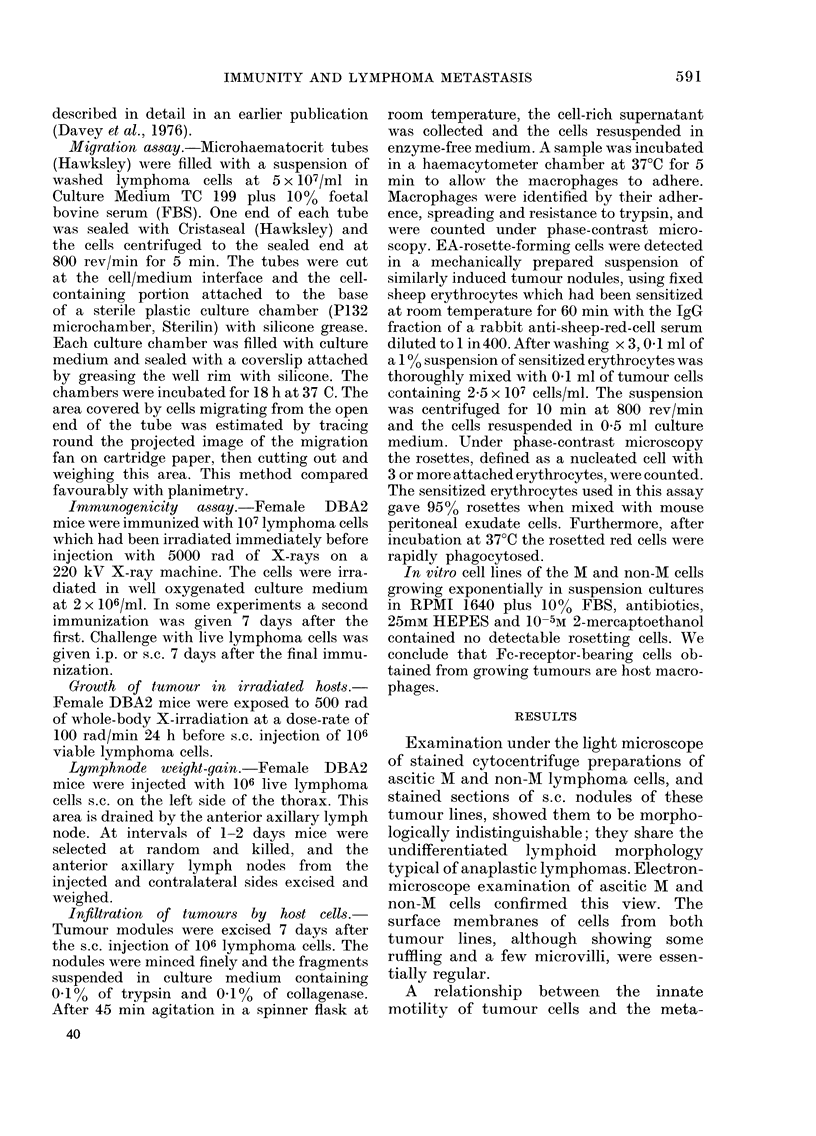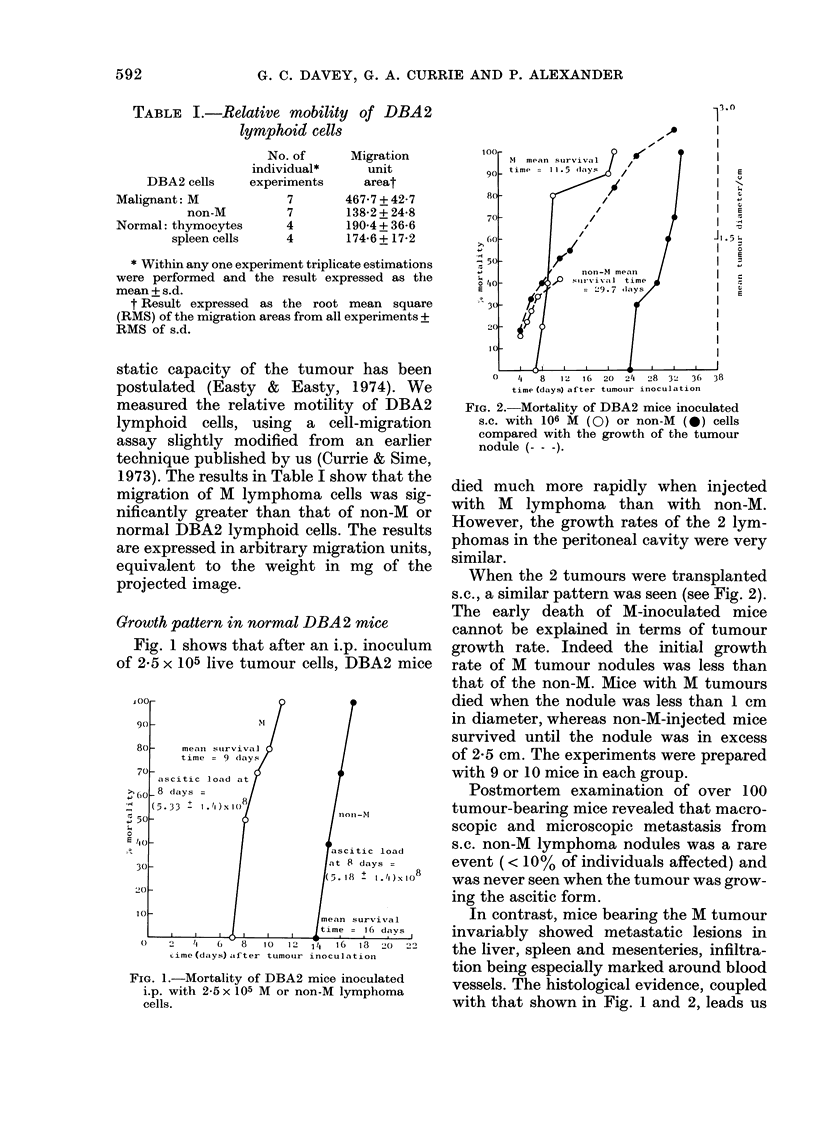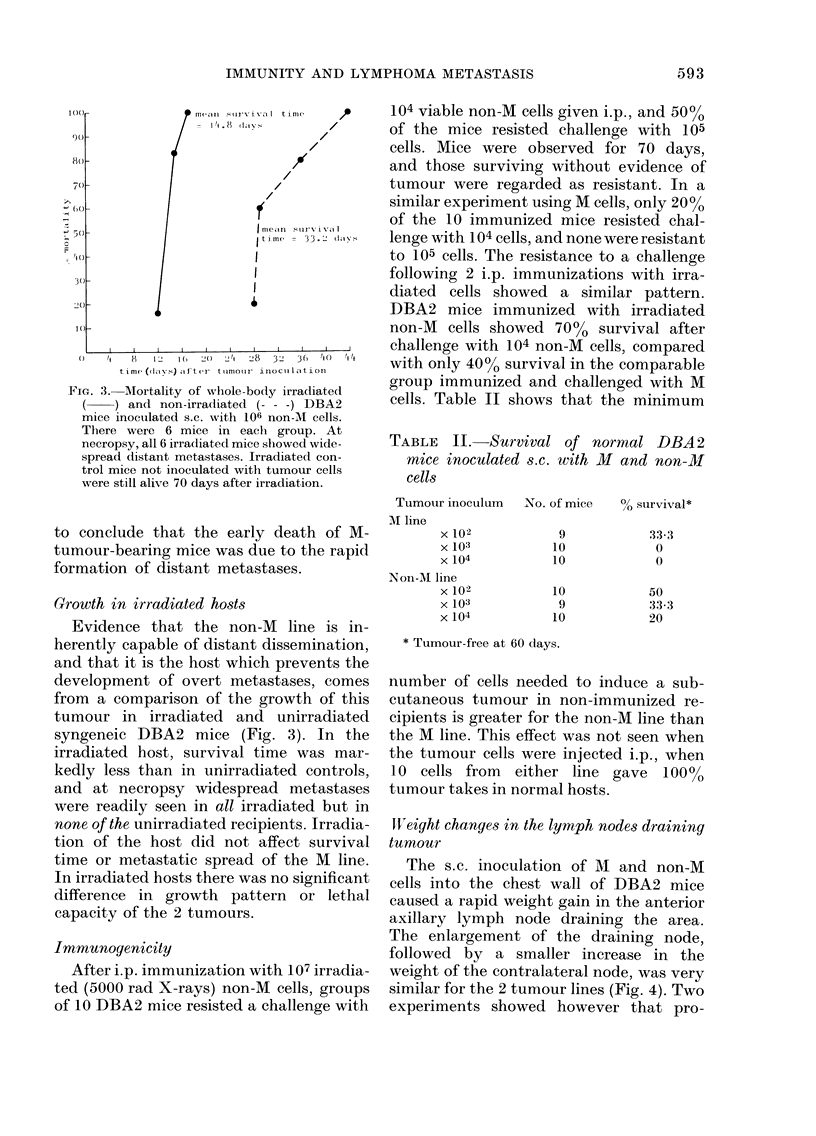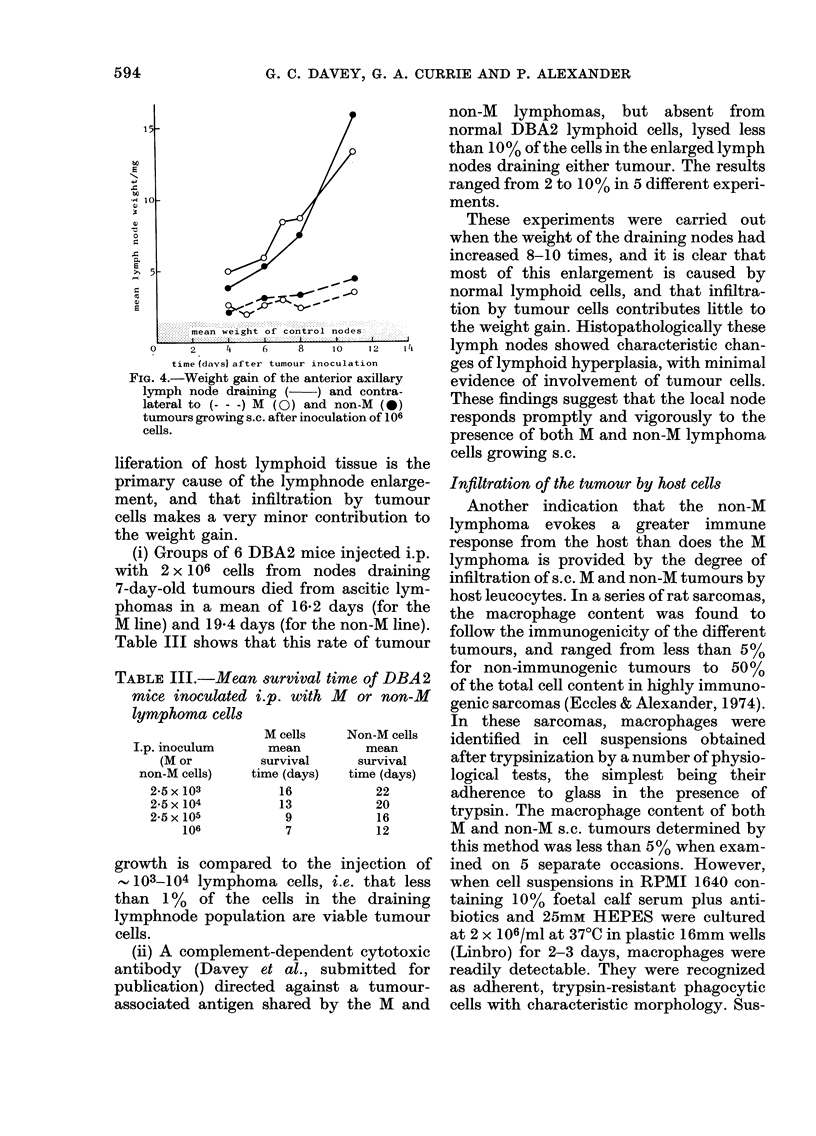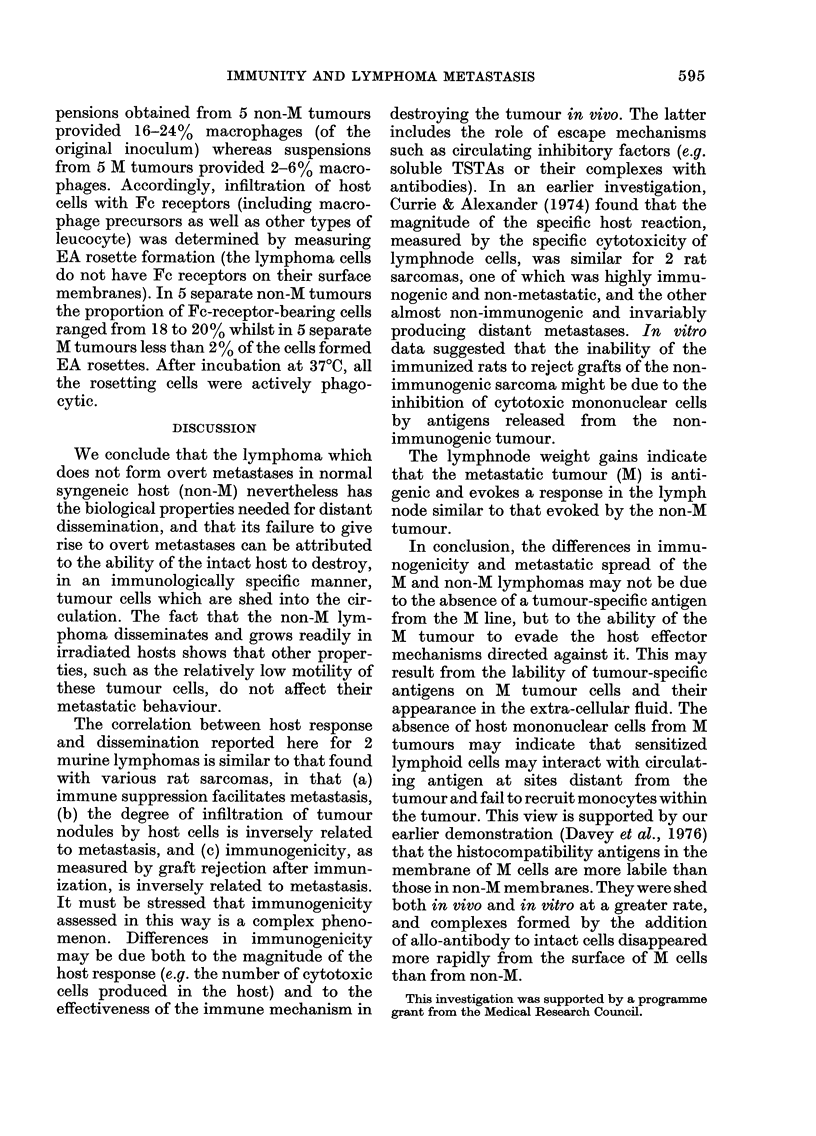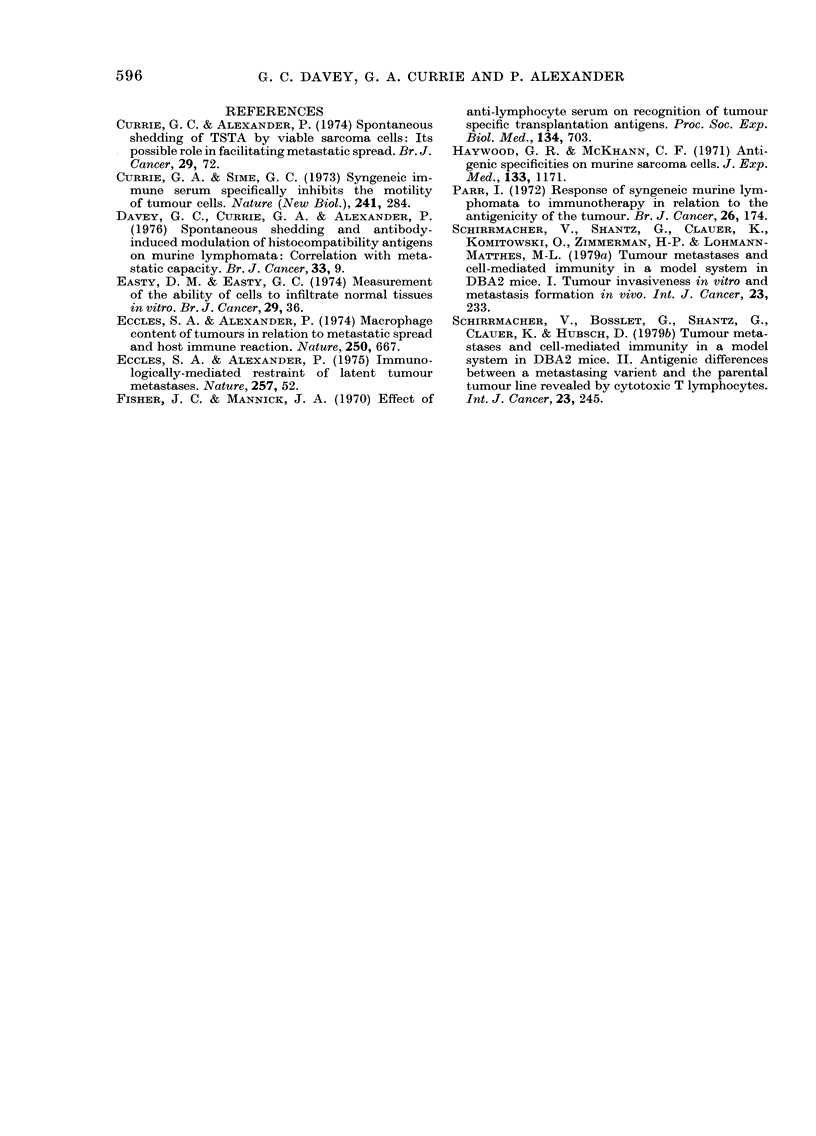# Immunity as the predominant factor determining metastasis by murine lymphomas.

**DOI:** 10.1038/bjc.1979.224

**Published:** 1979-10

**Authors:** G. C. Davey, G. A. Currie, P. Alexander

## Abstract

The metastatic behaviour of the L5178E (non-M) lymphoma and a highly metastatic subline L51787ES (M) were studied in syngeneic DBA2 mice. The non-M tumour rarely metastasizes in intact syngeneic mice, but produces extensive and rapidly lethal metastases when implanted into irradiated recipients. The metastatic behaviour of the M subline is unaffected by irradiation of the host. By conventional transplantation criteria, the non-M tumour is more immunogenic than the M subline. Both tumours, however, produce similar responses in a lymphnode weight-gain assay. Host-cell infiltration of the tumours growing s.c. is much greater in the non-M than the M, the infiltrating cells being Fc-receptor-positive and maturing into macrophages after 2 days in vitro. Although spontaneous in vitro motility of the M cells is much greater than that of the non-M, the metastatic behaviour of the tumours is clearly determined by host immunological responses.


Br. J. Cancer (1979) **40**, 590

### IMMUNITY AS THE PREDOMINANT FACTOR DETERMINING METASTASIS BY MURINE LYMPHOMAS

G. C. DAVEY, G. A. CURRIE AND P. ALEXANDER

*From the Division of Tumour Immunology, Chester Beatty Research Institute, Belmont, Sutton, Surrey*

Received 2 February 1979 Accepted 20 June 1979

**Summary.—The metastatic behaviour of the L5178E (non-M) lymphoma and a highly metastatic subline L51787ES (M) were studied in syngeneic DBA2 mice. The non-M tumour rarely metastasizes in intact syngeneic mice, but produces extensive and rapidly lethal metastases when implanted into irradiated recipients. The metastatic behaviour of the M subline is unaffected by irradiation of the host. By conventional transplantation criteria, the non-M tumour is more immunogenic than the M subline. Both tumours, however, produce similar responses in a lymphnode weight-gain assay. Host-cell infiltration of the tumours growing s.c. is much greater in the non-M than the M, the infiltrating cells being Fc-receptor-positive and maturing into macrophages after 2 days *in vitro*.**

**Although spontaneous *in vitro* motility of the M cells is much greater than that of the non-M, the metastatic behaviour of the tumours is clearly determined by host immunological responses.**

Two LINES of evidence suggest that an effective host immune response to tumour-specific transplantation-type antigens (TSTA) is a key factor in determining the metastatic behaviour of experimental sarcomas.

(i) The incidence of distant metastases is increased if the sarcomas are grown in rodents immunosuppressed by whole-body radiation (Eccles & Alexander, 1974), antilymphocyte serum (Fisher & Mannick, 1970) or selectively deprived of T cells (Eccles & Alexander, 1974, 1975).

(ii) There is an inverse correlation between the incidence of metastasis and immunogenicity (Haywood & McKhann, 1971; Eccles & Alexander, 1974).

We now show that both lines of evidence can be obtained from studies of 2 murine lymphomas. The original tumour L5178Y/E metastasizes only rarely when transplanted into its host of origin (DBA2 mice) and will be referred to as the "non-M" line. A variant of this lymphoma which arose spontaneously during routine passage (Parr, 1972) metastasizes widely, and will be referred to as the "M" line.

Schirrmacher *et al.* (1979*a*, *b*) examined these same tumours and have shown that the M cells (which they renamed ESb) differ from the non-M parent line (renamed Eb) in that they have more microvilli, show an increased capacity to adhere to normal tissues, and in an *in vitro* assay show increased invasiveness. They also demonstrated differences in immunogenicity and in antigenic specificity, and inferred that metastasis in this model is a complex multifactorial event. We now present evidence that host immunity is the paramount factor in determining metastatic behaviour in this model, and that unrelated features of the cells, such as adhesiveness, invasiveness and motility, play little or no role.

### MATERIALS AND METHODS

*Tumours.*—The origin and characteristics of the 2 lymphomas studied have been described in detail in an earlier publication (Davey *et al.*, 1976).

*Migration assay.*—Microhaematocrit tubes (Hawksley) were filled with a suspension of washed lymphoma cells at $5 \times 10^7$/ml in Culture Medium TC 199 plus 10% foetal bovine serum (FBS). One end of each tube was sealed with Cristaseal (Hawksley) and the cells centrifuged to the sealed end at 800 rev/min for 5 min. The tubes were cut at the cell/medium interface and the cell-containing portion attached to the base of a sterile plastic culture chamber (P132 microchamber, Sterilin) with silicone grease. Each culture chamber was filled with culture medium and sealed with a coverslip attached by greasing the well rim with silicone. The chambers were incubated for 18 h at 37 C. The area covered by cells migrating from the open end of the tube was estimated by tracing round the projected image of the migration fan on cartridge paper, then cutting out and weighing this area. This method compared favourably with planimetry.

*Immunogenicity assay.*—Female DBA2 mice were immunized with $10^7$ lymphoma cells which had been irradiated immediately before injection with 5000 rad of X-rays on a 220 kV X-ray machine. The cells were irradiated in well oxygenated culture medium at $2 \times 10^6$/ml. In some experiments a second immunization was given 7 days after the first. Challenge with live lymphoma cells was given i.p. or s.c. 7 days after the final immunization.

*Growth of tumour in irradiated hosts.*—Female DBA2 mice were exposed to 500 rad of whole-body X-irradiation at a dose-rate of 100 rad/min 24 h before s.c. injection of $10^6$ viable lymphoma cells.

*Lymphnode weight-gain.*—Female DBA2 mice were injected with $10^6$ live lymphoma cells s.c. on the left side of the thorax. This area is drained by the anterior axillary lymph node. At intervals of 1–2 days mice were selected at random and killed, and the anterior axillary lymph nodes from the injected and contralateral sides excised and weighed.

*Infiltration of tumours by host cells.*—Tumour modules were excised 7 days after the s.c. injection of $10^6$ lymphoma cells. The nodules were minced finely and the fragments suspended in culture medium containing 0·1% of trypsin and 0·1% of collagenase. After 45 min agitation in a spinner flask at room temperature, the cell-rich supernatant was collected and the cells resuspended in enzyme-free medium. A sample was incubated in a haemacytometer chamber at 37°C for 5 min to allow the macrophages to adhere. Macrophages were identified by their adherence, spreading and resistance to trypsin, and were counted under phase-contrast microscopy. EA-rosette-forming cells were detected in a mechanically prepared suspension of similarly induced tumour nodules, using fixed sheep erythrocytes which had been sensitized at room temperature for 60 min with the IgG fraction of a rabbit anti-sheep-red-cell serum diluted to 1 in 400. After washing $\times 3$, 0·1 ml of a 1% suspension of sensitized erythrocytes was thoroughly mixed with 0·1 ml of tumour cells containing $2{\cdot}5 \times 10^7$ cells/ml. The suspension was centrifuged for 10 min at 800 rev/min and the cells resuspended in 0·5 ml culture medium. Under phase-contrast microscopy the rosettes, defined as a nucleated cell with 3 or more attached erythrocytes, were counted. The sensitized erythrocytes used in this assay gave 95% rosettes when mixed with mouse peritoneal exudate cells. Furthermore, after incubation at 37°C the rosetted red cells were rapidly phagocytosed.

*In vitro* cell lines of the M and non-M cells growing exponentially in suspension cultures in RPMI 1640 plus 10% FBS, antibiotics, 25mM HEPES and $10^{-5}$M 2-mercaptoethanol contained no detectable rosetting cells. We conclude that Fc-receptor-bearing cells obtained from growing tumours are host macrophages.

### RESULTS

Examination under the light microscope of stained cytocentrifuge preparations of ascitic M and non-M lymphoma cells, and stained sections of s.c. nodules of these tumour lines, showed them to be morphologically indistinguishable; they share the undifferentiated lymphoid morphology typical of anaplastic lymphomas. Electron-microscope examination of ascitic M and non-M cells confirmed this view. The surface membranes of cells from both tumour lines, although showing some ruffling and a few microvilli, were essentially regular.

A relationship between the innate motility of tumour cells and the meta-

TABLE I.—*Relative mobility of DBA2 lymphoid cells*

| DBA2 cells | No. of individual* experiments | Migration unit area† |
|---|---|---|
| Malignant: M | 7 | 467·7 ± 42·7 |
| non-M | 7 | 138·2 ± 24·8 |
| Normal: thymocytes | 4 | 190·4 ± 36·6 |
| spleen cells | 4 | 174·6 ± 17·2 |

* Within any one experiment triplicate estimations were performed and the result expressed as the mean ± s.d.

† Result expressed as the root mean square (RMS) of the migration areas from all experiments ± RMS of s.d.

static capacity of the tumour has been postulated (Easty & Easty, 1974). We measured the relative motility of DBA2 lymphoid cells, using a cell-migration assay slightly modified from an earlier technique published by us (Currie & Sime, 1973). The results in Table I show that the migration of M lymphoma cells was significantly greater than that of non-M or normal DBA2 lymphoid cells. The results are expressed in arbitrary migration units, equivalent to the weight in mg of the projected image.

*Growth pattern in normal DBA2 mice*

Fig. 1 shows that after an i.p. inoculum of $2·5 \times 10^5$ live tumour cells, DBA2 mice

FIG. 1.—Mortality of DBA2 mice inoculated i.p. with $2·5 \times 10^5$ M or non-M lymphoma cells.

FIG. 2.—Mortality of DBA2 mice inoculated s.c. with $10^6$ M (○) or non-M (●) cells compared with the growth of the tumour nodule (- - -).

died much more rapidly when injected with M lymphoma than with non-M. However, the growth rates of the 2 lymphomas in the peritoneal cavity were very similar.

When the 2 tumours were transplanted s.c., a similar pattern was seen (see Fig. 2). The early death of M-inoculated mice cannot be explained in terms of tumour growth rate. Indeed the initial growth rate of M tumour nodules was less than that of the non-M. Mice with M tumours died when the nodule was less than 1 cm in diameter, whereas non-M-injected mice survived until the nodule was in excess of 2·5 cm. The experiments were prepared with 9 or 10 mice in each group.

Postmortem examination of over 100 tumour-bearing mice revealed that macroscopic and microscopic metastasis from s.c. non-M lymphoma nodules was a rare event (< 10% of individuals affected) and was never seen when the tumour was growing the ascitic form.

In contrast, mice bearing the M tumour invariably showed metastatic lesions in the liver, spleen and mesenteries, infiltration being especially marked around blood vessels. The histological evidence, coupled with that shown in Fig. 1 and 2, leads us

FIG. 3.—Mortality of whole-body irradiated (——) and non-irradiated (- - -) DBA2 mice inoculated s.c. with $10^6$ non-M cells. There were 6 mice in each group. At necropsy, all 6 irradiated mice showed widespread distant metastases. Irradiated control mice not inoculated with tumour cells were still alive 70 days after irradiation.

to conclude that the early death of M-tumour-bearing mice was due to the rapid formation of distant metastases.

*Growth in irradiated hosts*

Evidence that the non-M line is inherently capable of distant dissemination, and that it is the host which prevents the development of overt metastases, comes from a comparison of the growth of this tumour in irradiated and unirradiated syngeneic DBA2 mice (Fig. 3). In the irradiated host, survival time was markedly less than in unirradiated controls, and at necropsy widespread metastases were readily seen in *all* irradiated but in *none of the* unirradiated recipients. Irradiation of the host did not affect survival time or metastatic spread of the M line. In irradiated hosts there was no significant difference in growth pattern or lethal capacity of the 2 tumours.

*Immunogenicity*

After i.p. immunization with $10^7$ irradiated (5000 rad X-rays) non-M cells, groups of 10 DBA2 mice resisted a challenge with $10^4$ viable non-M cells given i.p., and 50% of the mice resisted challenge with $10^5$ cells. Mice were observed for 70 days, and those surviving without evidence of tumour were regarded as resistant. In a similar experiment using M cells, only 20% of the 10 immunized mice resisted challenge with $10^4$ cells, and none were resistant to $10^5$ cells. The resistance to a challenge following 2 i.p. immunizations with irradiated cells showed a similar pattern. DBA2 mice immunized with irradiated non-M cells showed 70% survival after challenge with $10^4$ non-M cells, compared with only 40% survival in the comparable group immunized and challenged with M cells. Table II shows that the minimum

TABLE II.—*Survival of normal DBA2 mice inoculated s.c. with M and non-M cells*

| Tumour inoculum | No. of mice | % survival* |
|---|---|---|
| M line | | |
| $\times 10^2$ | 9 | 33·3 |
| $\times 10^3$ | 10 | 0 |
| $\times 10^4$ | 10 | 0 |
| Non-M line | | |
| $\times 10^2$ | 10 | 50 |
| $\times 10^3$ | 9 | 33·3 |
| $\times 10^4$ | 10 | 20 |

* Tumour-free at 60 days.

number of cells needed to induce a subcutaneous tumour in non-immunized recipients is greater for the non-M line than the M line. This effect was not seen when the tumour cells were injected i.p., when 10 cells from either line gave 100% tumour takes in normal hosts.

*Weight changes in the lymph nodes draining tumour*

The s.c. inoculation of M and non-M cells into the chest wall of DBA2 mice caused a rapid weight gain in the anterior axillary lymph node draining the area. The enlargement of the draining node, followed by a smaller increase in the weight of the contralateral node, was very similar for the 2 tumour lines (Fig. 4). Two experiments showed however that pro-

FIG. 4.—Weight gain of the anterior axillary lymph node draining (——) and contralateral to (- - -) M (○) and non-M (●) tumours growing s.c. after inoculation of $10^6$ cells.

liferation of host lymphoid tissue is the primary cause of the lymphnode enlargement, and that infiltration by tumour cells makes a very minor contribution to the weight gain.

(i) Groups of 6 DBA2 mice injected i.p. with $2 \times 10^6$ cells from nodes draining 7-day-old tumours died from ascitic lymphomas in a mean of 16·2 days (for the M line) and 19·4 days (for the non-M line). Table III shows that this rate of tumour

TABLE III.—*Mean survival time of DBA2 mice inoculated i.p. with M or non-M lymphoma cells*

| I.p. inoculum (M or non-M cells) | M cells mean survival time (days) | Non-M cells mean survival time (days) |
|---|---|---|
| $2{\cdot}5 \times 10^3$ | 16 | 22 |
| $2{\cdot}5 \times 10^4$ | 13 | 20 |
| $2{\cdot}5 \times 10^5$ | 9 | 16 |
| $10^6$ | 7 | 12 |

growth is compared to the injection of $\sim 10^3$–$10^4$ lymphoma cells, *i.e.* that less than 1% of the cells in the draining lymphnode population are viable tumour cells.

(ii) A complement-dependent cytotoxic antibody (Davey *et al.*, submitted for publication) directed against a tumour-associated antigen shared by the M and non-M lymphomas, but absent from normal DBA2 lymphoid cells, lysed less than 10% of the cells in the enlarged lymph nodes draining either tumour. The results ranged from 2 to 10% in 5 different experiments.

These experiments were carried out when the weight of the draining nodes had increased 8–10 times, and it is clear that most of this enlargement is caused by normal lymphoid cells, and that infiltration by tumour cells contributes little to the weight gain. Histopathologically these lymph nodes showed characteristic changes of lymphoid hyperplasia, with minimal evidence of involvement of tumour cells. These findings suggest that the local node responds promptly and vigorously to the presence of both M and non-M lymphoma cells growing s.c.

*Infiltration of the tumour by host cells*

Another indication that the non-M lymphoma evokes a greater immune response from the host than does the M lymphoma is provided by the degree of infiltration of s.c. M and non-M tumours by host leucocytes. In a series of rat sarcomas, the macrophage content was found to follow the immunogenicity of the different tumours, and ranged from less than 5% for non-immunogenic tumours to 50% of the total cell content in highly immunogenic sarcomas (Eccles & Alexander, 1974). In these sarcomas, macrophages were identified in cell suspensions obtained after trypsinization by a number of physiological tests, the simplest being their adherence to glass in the presence of trypsin. The macrophage content of both M and non-M s.c. tumours determined by this method was less than 5% when examined on 5 separate occasions. However, when cell suspensions in RPMI 1640 containing 10% foetal calf serum plus antibiotics and 25mM HEPES were cultured at $2 \times 10^6$/ml at 37°C in plastic 16mm wells (Linbro) for 2–3 days, macrophages were readily detectable. They were recognized as adherent, trypsin-resistant phagocytic cells with characteristic morphology. Suspensions obtained from 5 non-M tumours provided 16–24% macrophages (of the original inoculum) whereas suspensions from 5 M tumours provided 2–6% macrophages. Accordingly, infiltration of host cells with Fc receptors (including macrophage precursors as well as other types of leucocyte) was determined by measuring EA rosette formation (the lymphoma cells do not have Fc receptors on their surface membranes). In 5 separate non-M tumours the proportion of Fc-receptor-bearing cells ranged from 18 to 20% whilst in 5 separate M tumours less than 2% of the cells formed EA rosettes. After incubation at 37°C, all the rosetting cells were actively phagocytic.

### DISCUSSION

We conclude that the lymphoma which does not form overt metastases in normal syngeneic host (non-M) nevertheless has the biological properties needed for distant dissemination, and that its failure to give rise to overt metastases can be attributed to the ability of the intact host to destroy, in an immunologically specific manner, tumour cells which are shed into the circulation. The fact that the non-M lymphoma disseminates and grows readily in irradiated hosts shows that other properties, such as the relatively low motility of these tumour cells, do not affect their metastatic behaviour.

The correlation between host response and dissemination reported here for 2 murine lymphomas is similar to that found with various rat sarcomas, in that (a) immune suppression facilitates metastasis, (b) the degree of infiltration of tumour nodules by host cells is inversely related to metastasis, and (c) immunogenicity, as measured by graft rejection after immunization, is inversely related to metastasis. It must be stressed that immunogenicity assessed in this way is a complex phenomenon. Differences in immunogenicity may be due both to the magnitude of the host response (*e.g.* the number of cytotoxic cells produced in the host) and to the effectiveness of the immune mechanism in destroying the tumour *in vivo*. The latter includes the role of escape mechanisms such as circulating inhibitory factors (*e.g.* soluble TSTAs or their complexes with antibodies). In an earlier investigation, Currie & Alexander (1974) found that the magnitude of the specific host reaction, measured by the specific cytotoxicity of lymphnode cells, was similar for 2 rat sarcomas, one of which was highly immunogenic and non-metastatic, and the other almost non-immunogenic and invariably producing distant metastases. *In vitro* data suggested that the inability of the immunized rats to reject grafts of the non-immunogenic sarcoma might be due to the inhibition of cytotoxic mononuclear cells by antigens released from the non-immunogenic tumour.

The lymphnode weight gains indicate that the metastatic tumour (M) is antigenic and evokes a response in the lymph node similar to that evoked by the non-M tumour.

In conclusion, the differences in immunogenicity and metastatic spread of the M and non-M lymphomas may not be due to the absence of a tumour-specific antigen from the M line, but to the ability of the M tumour to evade the host effector mechanisms directed against it. This may result from the lability of tumour-specific antigens on M tumour cells and their appearance in the extra-cellular fluid. The absence of host mononuclear cells from M tumours may indicate that sensitized lymphoid cells may interact with circulating antigen at sites distant from the tumour and fail to recruit monocytes within the tumour. This view is supported by our earlier demonstration (Davey *et al.*, 1976) that the histocompatibility antigens in the membrane of M cells are more labile than those in non-M membranes. They were shed both *in vivo* and *in vitro* at a greater rate, and complexes formed by the addition of allo-antibody to intact cells disappeared more rapidly from the surface of M cells than from non-M.

This investigation was supported by a programme grant from the Medical Research Council.

### REFERENCES

Currie, G. C. & Alexander, P. (1974) Spontaneous shedding of TSTA by viable sarcoma cells: Its possible role in facilitating metastatic spread. *Br. J. Cancer*, **29**, 72.

Currie, G. A. & Sime, G. C. (1973) Syngeneic immune serum specifically inhibits the motility of tumour cells. *Nature* (*New Biol.*), **241**, 284.

Davey, G. C., Currie, G. A. & Alexander, P. (1976) Spontaneous shedding and antibody-induced modulation of histocompatibility antigens on murine lymphomata: Correlation with metastatic capacity. *Br. J. Cancer*, **33**, 9.

Easty, D. M. & Easty, G. C. (1974) Measurement of the ability of cells to infiltrate normal tissues *in vitro*. *Br. J. Cancer*, **29**, 36.

Eccles, S. A. & Alexander, P. (1974) Macrophage content of tumours in relation to metastatic spread and host immune reaction. *Nature*, **250**, 667.

Eccles, S. A. & Alexander, P. (1975) Immunologically-mediated restraint of latent tumour metastases. *Nature*, **257**, 52.

Fisher, J. C. & Mannick, J. A. (1970) Effect of anti-lymphocyte serum on recognition of tumour specific transplantation antigens. *Proc. Soc. Exp. Biol. Med.*, **134**, 703.

Haywood, G. R. & McKhann, C. F. (1971) Antigenic specificities on murine sarcoma cells. *J. Exp. Med.*, **133**, 1171.

Parr, I. (1972) Response of syngeneic murine lymphomata to immunotherapy in relation to the antigenicity of the tumour. *Br. J. Cancer*, **26**, 174.

Schirrmacher, V., Shantz, G., Clauer, K., Komitowski, O., Zimmerman, H-P. & Lohmann-Matthes, M-L. (1979*a*) Tumour metastases and cell-mediated immunity in a model system in DBA2 mice. I. Tumour invasiveness *in vitro* and metastasis formation *in vivo*. *Int. J. Cancer*, **23**, 233.

Schirrmacher, V., Bosslet, G., Shantz, G., Clauer, K. & Hubsch, D. (1979*b*) Tumour metastases and cell-mediated immunity in a model system in DBA2 mice. II. Antigenic differences between a metastasing varient and the parental tumour line revealed by cytotoxic T lymphocytes. *Int. J. Cancer*, **23**, 245.